# Identification of a *R2R3-MYB* gene regulating anthocyanin biosynthesis and relationships between its variation and flower color difference in lotus (*Nelumbo* Adans.)

**DOI:** 10.7717/peerj.2369

**Published:** 2016-09-01

**Authors:** Shan-Shan Sun, Paul F. Gugger, Qing-Feng Wang, Jin-Ming Chen

**Affiliations:** 1 Key Laboratory of Aquatic Botany and Watershed Ecology, Wuhan Botanical Garden, Chinese Academy of Sciences, Wuhan, China; 2 University of Chinese Academy of Sciences, Beijing, China; 3 Appalachian Laboratory, University of Maryland Center for Environmental Science, Frostburg, Maryland, USA

**Keywords:** Flower color, Anthocyanin, MYB5 transcription factor, *Nelumbo*

## Abstract

The lotus (Nelumbonaceae: *Nelumbo* Adans.) is a highly desired ornamental plant, comprising only two extant species, the sacred lotus (*N. nucifera* Gaerten.) with red flowers and the American lotus (*N. lutea* Willd.) with yellow flowers. Flower color is the most obvious difference of two species. To better understand the mechanism of flower color differentiation, the content of anthocyanins and the expression levels of four key structural genes (e.g., *DFR*, *ANS*, *UFGT* and *GST*) were analyzed in two species. Our results revealed that anthocyanins were detected in red flowers, not yellow flowers. Expression analysis showed that no transcripts of *GST* gene and low expression level of three *UFGT* genes were detected in yellow flowers. In addition, three regulatory genes (*NnMYB5*, *NnbHLH1* and *NnTTG1*) were isolated from red flowers and showed a high similarity to corresponding regulatory genes of other species. Sequence analysis of *MYB5*, *bHLH1* and *TTG1* in two species revealed striking differences in coding region and promoter region of *MYB5* gene. Population analysis identified three *MYB5* variants in *Nelumbo*: a functional allele existed in red flowers and two inactive forms existed in yellow flowers. This result revealed that there was an association between allelic variation in *MYB5* gene and flower color difference. Yeast two-hybrid experiments showed that NnMYB5 interacts with NnbHLH1, NlbHLH1 and NnTTG1, and NnTTG1 also interacts with NnbHLH1 and NlbHLH1. The over-expression of *NnMYB5* led to anthocyanin accumulation in immature seeds and flower stalks and up-regulation of expression of *TT19* in *Arabidopsis*. Therefore, NnMYB5 is a transcription activator of anthocyanin synthesis. This study helps to elucidate the function of *NnMYB5* and will contribute to clarify the mechanism of flower coloration and genetic engineering of flower color in lotus.

## Introduction

In most species, flower color is attributed to the accumulation of anthocyanins (Quattrocchio et al., 2006). Anthocyanin are the major water-soluble pigments, produced by the flavonoid biosynthesis pathway, widely distributed in flowering plants and responsible for pink, purple, red, and blue of many flowers and fruits (Chandler & Tanaka, 2007; Wessinger & Rausher, 2014). Currently, all structural genes involved in the anthocyanin synthesis have been well characterized in many species (Schwinn & Davies, 2004; Grotewold, 2006; Guo et al., 2014). Anthocyanins are synthesized in the cytosol, and are not stabilized until they are transported into vacuole (Kitamura, Shikazono & Tanaka, 2004; Gomez et al., 2011). *Glutathione S-transferase* (*GST*) genes are thought to be involved in anthocyanins transport in many species, such as *Arabidopsis TT19*, maize *Bz2*, petunia *AN9*, grape *VvGST1 and VvGST4*, cyclamen *CkmGST3* and peach *Riant* (Marrs et al., 1995; Alfenito et al., 1998; Kitamura, Shikazono & Tanaka, 2004; Conn et al., 2008; Kitamura et al., 2012; Cheng et al., 2015).

The structural genes are highly conserved among species (Holton & Cornish, 1995). Regulation of structural genes at the level of transcription appears to be the major mechanism that leads to the diversity of flower color in plants (Quattrocchio et al., 1998; Yamagishi et al., 2010). In all species studied to date, the anthocyanin biosynthesis is regulated by a complex of R2R3-MYB and basic helix-loop-helix (bHLH) transcription factors (TF) as well as WD40 proteins (Broun, 2005; Koes, Verweij & Quattrocchio, 2005; Petroni & Tonelli, 2011). Among them, the R2R3-MYB proteins can directly bind to the promoters of structural genes to activate their expression with the help of bHLH and WD40 proteins and are thought to be the most key TF (Takos et al., 2006; Zhang, Butelli & Martin, 2014; Tian et al., 2015). Anthocyanin-regulating *R2R3-MYB* genes were isolated from many ornamental plants and economic crops, such as grape *VvMybA1*, tomato *ANT1*, apple *MdMYB10*, gerbera *GMYB10*, orchid *OgMYB1*, gentian *GtMYB3*, pear *PyMYB10*, lily *LhMYB12*, crabapple *McMYB10* and peach *PpMYB10.1* (Kobayashi et al., 2002; Mathews et al., 2003; Espley et al., 2007; Chiou & Yeh, 2008; Laitinen et al., 2008; Nakatsuka et al., 2008; Feng et al., 2010; Yamagishi, Yoshida & Nakayama, 2012; Tian et al., 2015; Tuan et al., 2015). Examples of the interaction between anthocyanin-related MYBs and bHLHs include *Zea mays* ZmC1 and ZmB, gerbera GMYC1 and GMYB10, *Arabidopsis* PAP1, PAP2, MYB113 & MYB114 and TT8, petunia AN2 and AN1, apple MdMYB10 and MdbHLH33, and litchi LcMYB1 and LcbHLH1, LcbHLH3 (Goff, Cone & Chandler, 1992; Elomaa et al., 2003; Zimmermann et al., 2004; Quattrocchio et al., 2006; Espley et al., 2007; Lai et al., 2016).

The lotus (Nelumbonaceae: *Nelumbo* Adans.) is an ancient aquatic plants with large, showy and fragrant flowers, comprising only two extant species, the sacred lotus (*N. nucifera* Gaerten.) and the American lotus (*N. lutea* Willd.). *N. nucifera* is native to Asia and is the national flower of India; *N. lutea* is native to North America (Tian et al., 2008). Although these two species are allopatric, their hybrids are fertile. Among external morphologies (e.g., plant size, leaf shape, petal shape and color), petal color is the most obvious difference that distinguishes these two species of *Nelumbo*. The petal color of the wild sacred lotus is mainly red, whereas that of the American lotus is yellow. In Asia, the sacred lotus, *N. nucifera*, has been cultivated for more than 7,000 years for its beautiful flower as well as medicinal and cooking uses (Zhang, Chen & Wang, 2011). Lotus is one of the top ten traditional garden flowers in China, and over 800 cultivars are now under cultivation (Zhang, Chen & Wang, 2011). The petals of lotus cultivars possess a variety of bright colors including red, pink, white with red borders, plain white, and yellow.

So far, the relationship between flower color and pigments composition and content was elucidated among lotus cultivars using HPLC (Katori et al., 2002; Chen et al., 2013; Deng et al., 2013). The results showed that the red and pink flower cultivars contained all types of anthocyanins except for pelargonidin, while no anthocyanins were detected in white and yellow flower cultivars; flavones and flavonols were detected in all cultivars and small amounts of xanthophylls and β-carotene were detected in some yellow cultivars. Based on the analyses of metabolomic analysis, a putative flavonoid biosynthetic pathway was proposed in lotus (Chen et al., 2013). The whole-genome sequence of lotus has been reported, which facilitate the functional genomics studies in this species (Ming et al., 2013). The flower color difference between red and white lotus cultivars may be attributed to the different methylation intensities on the promoter sequences of the *ANS* gene (Deng et al., 2015). Although these studies have provided valuable insights into the mechanism of flower coloration in lotus cultivars, the regulation mechanism of anthocyanin biosynthesis and the cause of flower color difference between two species in *Nelumbo* remain unclear. In this study, regulatory genes were identified from lotus genome and analyzed in two lotus species. Three regulatory genes (*MYB5*, *bHLH1* and *TTG1*) were isolated from red flowers. The interaction among three regulatory proteins and the function of *NnMYB5* were tested.

## Materials and Methods

### Plant materials

The seeds of the wild lotus *N. nucifera* and *N. lutea* were collected from Yilan population of Heilongjiang province of China and Lake Jackson population of Florida of United States of America (USA), respectively. Seeds were germinated and grown at the Wuhan Botanical Garden (WBG), Chinese Academy of Sciences, under the same natural conditions. The flowers of these plants were collected in pre-anthesis while the petals were deeply pigmented. These flowers were immediately frozen with liquid nitrogen and stored at −80 °C for RNA extraction and anthocyanin extraction. The fresh leaves of each species were also collected and stored at −20 °C for DNA extraction.

To test whether there is a relationship between flower color difference of two species and regulatory genes, a total of 97 individuals from 16 wild lotus populations were further collected, including 49 individuals from eight populations of *N. nucifera* with red flower collected from China and Thailand and 48 individuals from eight populations of *N. lutea* with yellow flowers collected from USA (Table S1). For this analysis, fresh leaves of each individual were collected and dried immediately with silica gel and stored at room temperature. Voucher specimens from all populations were deposited in the herbarium of the WBG.

### Anthocyanin extraction and measurement

Total anthocyanins were extracted from the petals according to the methods described previously (Rahim, Busatto & Trainotti, 2014) and anthocyanin contents were measured using spectrophotometry according to the formula, Q_Anthocyanins_ = (A_530_ − 0.25 × A_657_) × M^−1^ (Mehrtens et al., 2005), where Q_Anthocyanins_ is the amount of anthocyanins, A_530_ and A_657_ is the absorption at 530 and 657 nm wavelength, respectively, and M is the fresh weight (g) of the tissues.

### DNA, RNA extraction and qRT-PCR analysis

Total RNA was isolated from petals using Quick RNA Isolation Kit for Polysaccharides and Polyphenolics-rich Plant (Zoman, Beijing, China). A DNA extraction kit (ComWin, Beijing, China) was used to extract DNA from leaves. Each RNA sample was treated with RNase-free DNase I (TaKaRa) to eliminate contaminating genomic DNA before the reverse transcription (RT) reaction. RT-PCR was performed according to the standard instructions of the PrimeScript RT Reagent Kit with gDNA Eraser (TaKaRa).

Considering a lot of flavones and flavonols in red and yellow flowers in lotus (Deng et al., 2013), which indicates that the upstream genes in the anthocyanin biosynthetic pathway are active, we only investigated the expression levels of downstream genes (*DFR*, *ANS*, *UFGT* and *GST*) by quantitative reverse transcription–PCR (qRT-PCR) and semi-quantitative RT-PCR (sqRT-PCR). qRT-PCR was conducted using StepOne Real-Time PCR System (Applied Biosystems, Foster City, CA, USA). A total reaction volume of 20 μL contained 10 μL of 2 × SYBR premix Ex Taq™ II (DRR082A, TaKaRa), 0.3 μM of each primer, about 100 ng of template cDNA. The amplification condition was as follows: incubation at 95 °C for 30 s, followed by 40 cycles of 95 °C for 3 s, 57 °C for 30 s and 72 °C for 30 s. A lotus actin gene (GenBank ID: EU131153) was used as a constitutive control. Gene relative expression levels of target genes was calculated by 2^−ΔΔCt^ comparative threshold cycle (Ct) method, and three replicates were performed. Primer sequences (Table S2) were designed on the whole genome data of *N. nucifera* (GenBank accession: AQOG00000000; Ming et al., 2013) and the whole-genome resequencing data of *N. lutea* (∼10× coverage depth; J. M. Chen, 2015, unpublished data).

### Identification of candidate R2R3-MYB, bHLH and WD40 transcription factors in the lotus genome

To identify the candidate R2R3-MYB, bHLH and WD40 proteins in the lotus genome, the R2R3 motif of *Arabidopsis* AtPAP1 (NP_176057.1) and the whole amino acid sequences of petunia PhAN11 (NP_197840.1) and *Arabidopsis* AtTTG1 (AAG25927.1) were used to tblastn against our local whole genome database of *N. nucifera*. The matched sequences with the E-value (Expect value) less than 1e−10 were used. Amino acid sequence alignments were performed using the ClustalW (Thompson, Higgins & Gibson, 1994). A phylogenetic tree was subsequently constructed according to the neighbor-joining (NJ) statistical method (Perrière & Gouy, 1996) using the program MEGA5 (Tamura et al., 2011). Confidence in the nodes was tested by performing 1,000 bootstrap replicates (Felsenstein, 1985).

Based on the result of our phylogenetic analyses, the specific primer pairs, such as MYBU1F/U1R, MYBU2F1/R1, MYBU2F2/R2 and MYBU3F/U3R for *MYB* genes and bHLH205F/1420R for *bHLH* gene, were designed in the conserved regions (Table S2) to determine which *MYB* genes and *bHLH* genes involved in anthocyanin synthesis were expressed in petals of *N. nucifera*. The red flowers with high content of anthocyanin were then chosen to isolate the *MYB* and *bHLH* genes. The PCR reactions were carried out using the specific primers designed above to amplify cDNAs of red flowers. The amplified fragments were then cloned and sequenced. A number of cDNAs encoding R2R3-MYB domains were obtained using the primer MYBU1F/U1R and all these cDNA clones showed highest identity to *NnMYB5*. Only one *bHLH* gene (thereafter named “*NnbHLH1*”) was detected in petals of *N. nucifera*. In addition, only one WD40 protein, named “*NnTTG1*,” was considered to possibly regulate anthocyanin biosynthesis in lotus according to our phylogenetic analyses. In order to study the regulatory gene involved in flower color, so in our subsequent analyses in *N. nucifera*, we only focused on the three regulatory genes (*NnMYB5, NnbHLH1* and *NnTTG1*) expressed in red flower.

### Cloning and sequencing of *MYB5*, *bHLH1* and *TTG1* in lotus

For two species in *Nelumbo*, the full-length cDNAs sequences and genomic sequences of *MYB5, bHLH1* and *TTG1* genes were amplified by using the primer pairs, MYBU1F/MYB5gR, bHLHgF/gR and TTG1gF/R, respectively. The promoter regions of *MYB5* in two species were also cloned using MYBpF/R primer. The details of primer sequences were provided (Table S2).

### Yeast two-hybrid analysis

To investigate protein interactions between NnMYB5 and NnbHLH1 or NlbHLH1 (obtained from *N. lutea*), between NnTTG1 and NnMYB5, and between NnTTG1 and NnbHLH1 or NlbHLH1, the Matchmaker Two-Hybrid System 3 (Clontech, Mountain View, CA, USA) was employed in the yeast two-hybrid (Y2H) assay. The full-length coding sequences of *NnMYB5*, *NnbHLH1*, *NlbHLH1* and *NnTTG1* were cloned into vector pGBK-T7, and then transformed into yeast strain Y2HGOLD according to manufacturer’s instructions (Yeastmaker, Clontech) to check self-activation. The open reading frame (ORF) sequences of *NnMYB5* and *NnTTG1* were cloned into the pGAD-T7 (with GAL4AD), and then transformed into yeast strain Y187. The primers used in Y2H system are shown (Table S2). After mating, yeast was plated onto SD–Trp–Leu plates. Positive colonies were validated by PCR amplification and then streaked on new SD–Trp–Leu and SD–Trp–Leu–His–Ade plates. Finally, positive colonies were transferred onto SD–Trp–Leu–His–Ade + X-α-Gal plates to further validate positive interaction between the two tested proteins.

### Transgenic plasmid construction and plant transformation

To confirm the function of *NnMYB5* gene in lotus, the genomic DNA of *NnMYB5* was amplified and cloned into the pCAMBIA1301 vector under the control of CaMV 35S promoter with the primers 5′-CGAGCTCATGGATGGTGGTTTGGGTTT-3′ (forward) and 5′-CGGGATCCTCAATAACTCCACCACCTATG-3′ (reverse).

The above recombinant construct was then transformed into *Agrobacterium tumefaciens* strain GV3101 and introduced into *Arabidopsis thaliana* WT (Col-0) plants using the floral dip method (Clough & Bent, 1998) to obtain *NnMYB5* over-expressing plants. *Arabidopsis* seeds were sterilized with 70% (v/v) ethyl alcohol, 10% (w/v) NaCl and deionized water. After stratification at 4 °C for 3 d in darkness, transgenic *Arabidopsis* seeds were selected on half-strength Murashige and Skoog (MS) media containing 30 mg/L Hygromycin. Hygromycin-resistant T1 seedlings were transferred to soil and grown at 23 °C in a growth chamber with a 16-h day length. T2 seedlings were sowed in soil. The nutrient solution was watered from below in pots with soil-grown plants twice every week. The transgenic plants were confirmed by PCR analyses. Additionally, the expression levels of *NnMYB5* and *TT19* (At5g17220) *TT19* gene were determined by semi-quantitative RT-PCR (sqRT-PCR) and the actin gene was used as an internal control. The primers used for sqRT-PCR are shown (Table S2).

### The analysis of relationships between flower color differences among two species and *MYB5* genes

To further test whether the flower color difference between red flowers and yellow flowers is related to *MYB5* genes, the genomic sequences of *MYB5* were amplified using the total DNA of 97 individuals from 16 wild populations of lotus (eight populations each species) and the primers 5′-GATAAGTGGAGAAGGCTATGACTGG-3′ (forward) and 5′-TCAATAACTCCACCACCTATGATTCA-3′ (reverse). PCR products were purified using the SanPrep Column PCR Product Purification Kit (Sangon, Shanghai, China) and were sequenced by Sangon Biotech. Co., Ltd. (Shanghai, China).

### Accession numbers

Sequence data used in this study can be retrieved from NCBI (http://www.ncbi.nlm.nih.gov/) under the following accession numbers: ZmC1 (AAK09327.1), ZmP1 (AAA19821.1), VvMYBA2 (BAD18978.1), VvMYBA1 (BAD18980.1), VvMYB5b (AAX51291.1), VvMYBA3 (BAD18979.1), VvMYBCs1 (AAS68190.1), SlANT1 (AAQ55181.1), PhODO1 (AAV98200.1), PhMYB3 (CAA78388.1), PhMYB2 (CAA78387.1), PhMYB1 (CAA78386.1), PhAN2 (AAF66727.1), PH4 (AAY51377.1), OsMYB4 (BAA23340.1), IpMYB1 (BAE94388.1), InMYB3 (BAE94710.1), InMYB2 (BAE94709.1), InMYB1 (BAE94389.1), GhBNLGHi233 (AAK19611.1), CaMYB (ABN11121.1), AtPAP1 (AAG42001.1), AtPAP2 (AAG42002.1), AmVENOSA (ABB83828.1), AmROSEA1 (ABB83826.1), AmROSEA2 (ABB83827.1), AmMIXTA (CAA55725.1); NnbHLH1 (KU198709), NnbHLH2 (XP_010278105.1), NnbHLH3 (XP_010255365.1), NnbHLH4 (XP_010241898.1), NnbHLH5 (XP_010273162.1), NnbHLH6 (XP_010275210.1), NnbHLH7 (XP_010242395.1), NnbHLH8 (XP_010256776.1), NnbHLH9 (XP_010267440.1), NnbHLH10 (XP_010252341.1), LhbHLH2 (BAE20058.1), PhAN1 (AAG25927.1), AtTT8 (NP_192720.2), InbHLH2 (BAE94394.1), ZmIN1 (AAB03841.1), OsRc (ABB17166.1), PfMYC-RP (BAA75513.1), PfF3G1 (BAC56998.1), OsRa (AAC49219.1), ZmLc (NP_001105339.1), PhJAF13 (AAC39455.1), InbHLH3 (BAE94395.1), InbHLH1 (BAE94393.1), GMYC1 (CAA07615.1), AtGL3 (NP_680372.1), AtEGL1 (NP_176552.1), AmDEL (AAA32663.1), AtMYC1 (BAA11933.1); NnTTG1 (XP_010251384.1), NnLWD1 (XP_010250423.1), NnLWD1-like (XP_010258659.1) and NnLWD1-like variant X1 (XP_010271596.1), ZmPAC1 (AAM76742), PhAN11 (AAC18914), PFDS (BAB58883), MtWD40-1 (ABW08112), IpWDR1 (BAE94396), InWDR1 (BAE94398), GhTTG1(AF336281.1), GhTTG3 (AAM95645), AtTTG1 (Q9XGN1).

## Results

### Anthocyanin content and expression analysis of *DFR*, *ANS, UFGT* and *GST* genes between red flowers and yellow flowers

**Figure 1:**
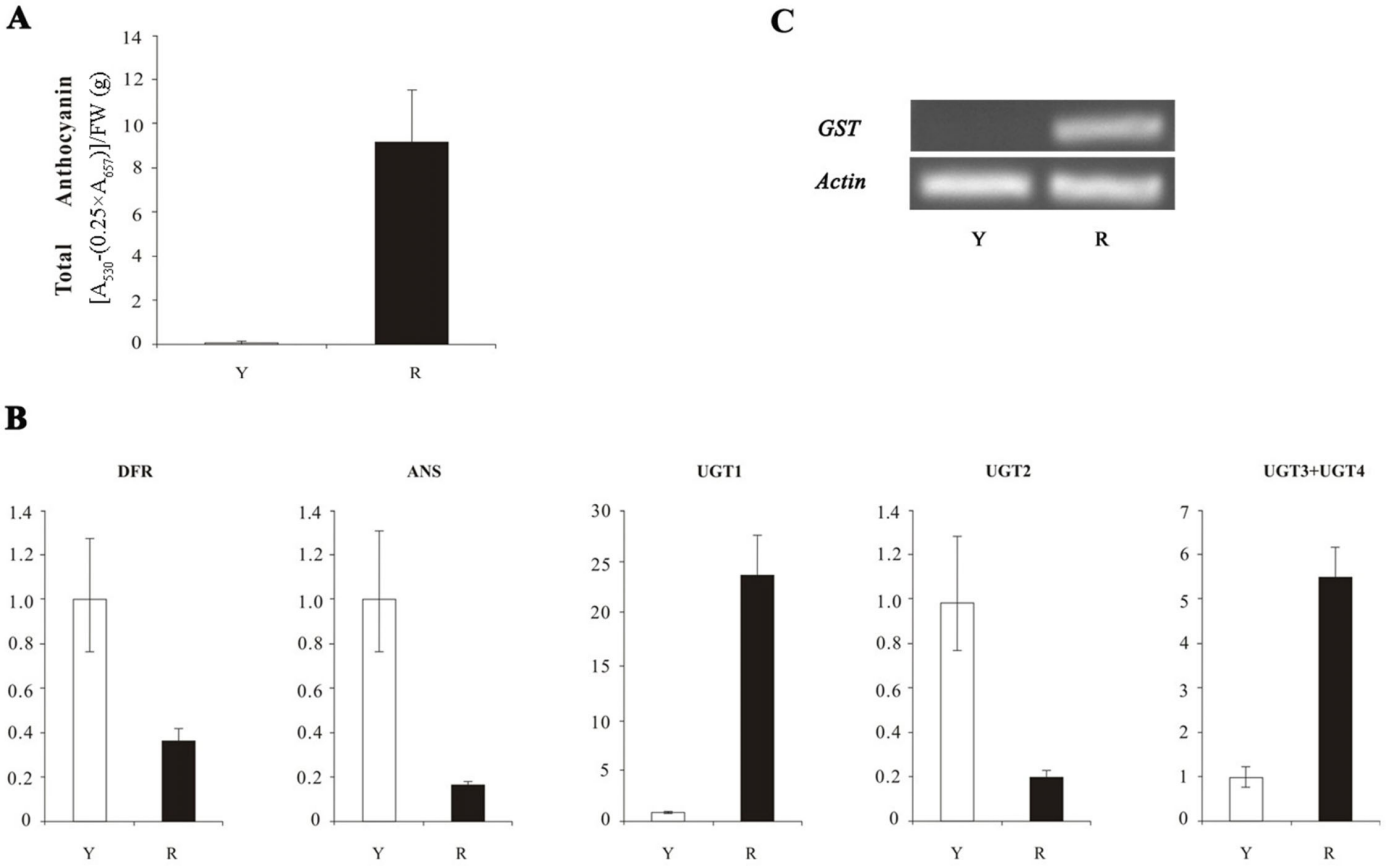
The anthocyanin content and the expression analyses of four structural genes (*DFR*, *ANS*, *UFGT* and *GST*) in *Nelumbo*. (A) Total anthocyanin content in petals in two species. Error bars are ± SD of the means of triplicates. (B) The expression profile of three structural genes (*DFR*, *ANS* and *UFGT*) in two species as determined by qRT-PCR. Due to high homology of *UGT3* and *UGT4*, the expression level of two genes was detected together by a pair of primers. (C) The transcription levels of *GST* in *N. lutea* and *N. nucifera* as determined by sqRT-PCR. Actin gene was used as internal control. Y, yellow petal in *Nelumbo lutea*; R, red petals in *N. nucifera*.

The result indicated a lot of anthocyanin in red flowers and no anthocyanin in yellow flowers (Fig. 1A). The qRT-PCR results showed that the expression of *DFR*, *ANS*, and *UGT2* in yellow petals was higher than that of in red petals (Fig. 1B), which was contradicted with anthocyanin content. The expression levels of three *UFGT* genes (*UGT1*, *UGT3* and *UGT4*) in yellow petals of *N. lutea* were lower than in red petals of *N. nucifera* and no transcripts of *GSTF11* gene were detected in *N. lutea* with yellow flowers, which was consistent with anthocyanin content (Fig. 1C).

### Identification of R2R3-MYBs, bHLHs and WD40 transcription factors in the lotus genome

**Figure 2:**
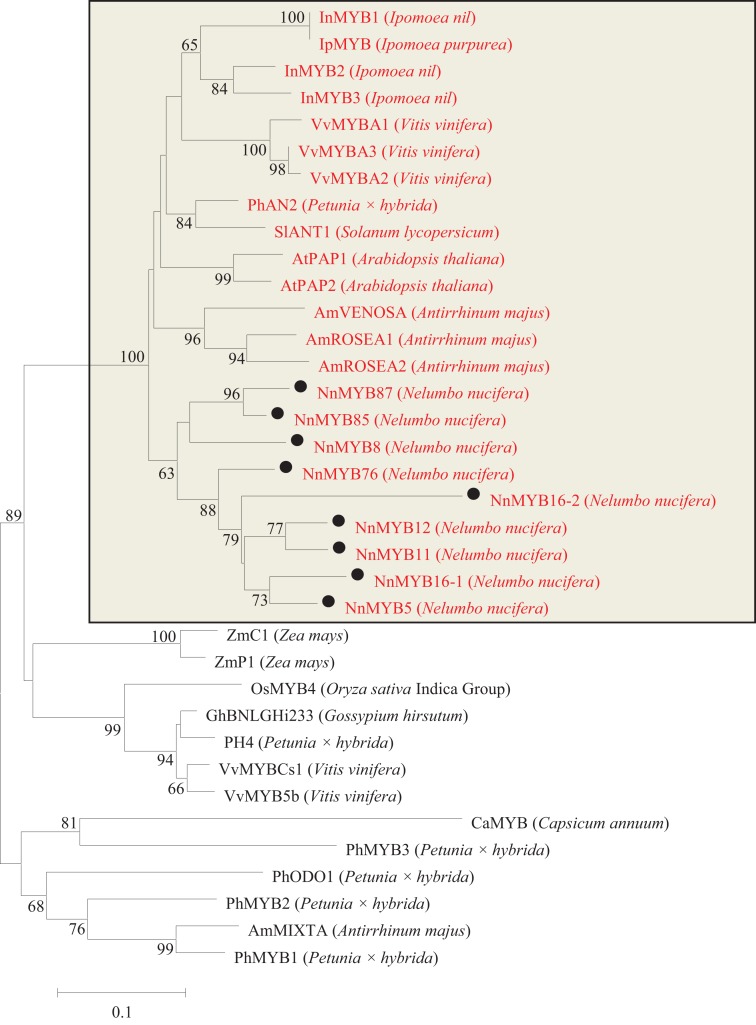
Phylogentic relationships of MYB proteins from lotus and other species. Neighbor-joining tree showing relationships between the nine lotus MYB proteins with MYBs of other species. Numbers close to the nodes indicate bootstrap values, with only values above 50 shown, and scale shows 0.1 amino acid substitutions per site. The deduced amino acid sequences were retrieved from NCBI. Black circles represent lotus MYB proteins that were clustered with MYBs of other species known to be involved in the regulation of anthocyanin biosynthesis. All MYBs involved in anthocyanin synthesis were located in the rectangle and labeled as red letters.

By local blast search, 95 *MYBs*, 10 *bHLHs* and 17 *WD40s* were found in the genome database of *N. nucifera* (Table S3). Based on the highly conserved R2R3 motifs in plants, a total of 92 R2R3 motifs of R2R3-MYBs were identified in the genome database of *N. nucifera*. Phylogenetic analysis revealed that nine MYB TFs in *N. nucifera* were clustered with the known MYB TFs involved in anthocyanin biosynthesis in other species, e.g., *Arabidopsis* AtPAP1 and AtPAP2, petunia PhAN2, grape VvMYBA1-A3, *Ipomoea nil* InMYB2, and snapdragon AmROSEA1-2 (Figs. 2 and S1).

**Figure 3:**
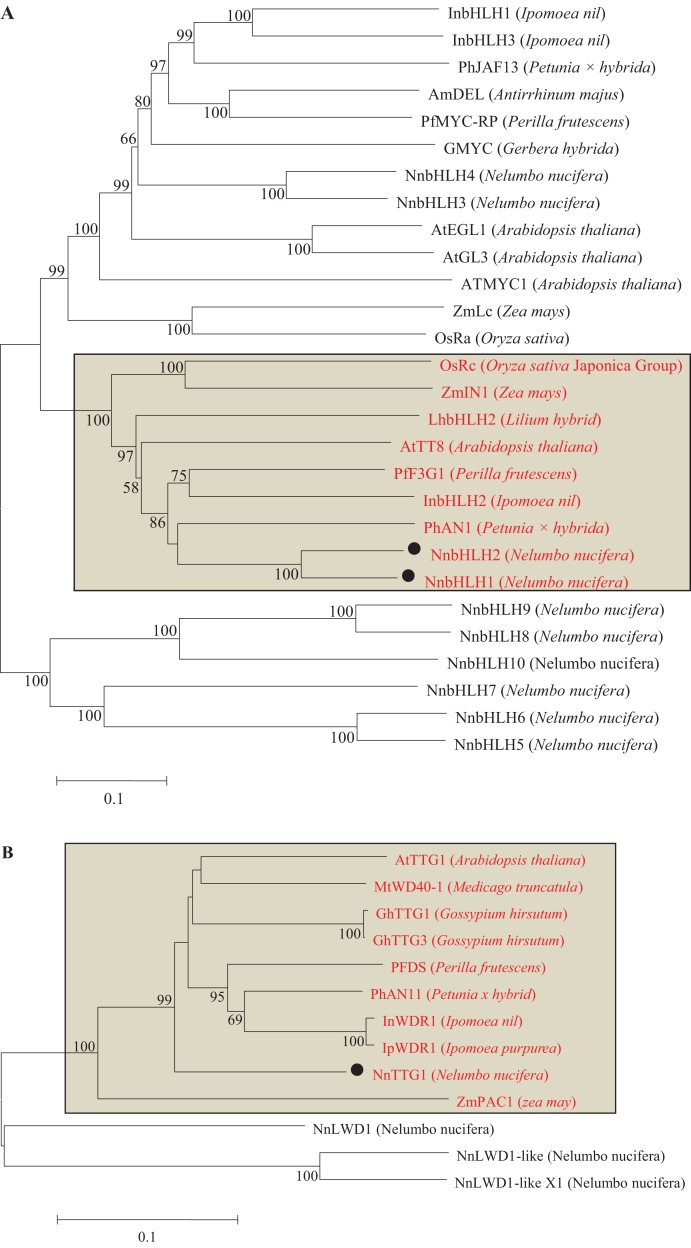
Phylogentic relationships of bHLH and WD40 proteins from lotus and other species. (A) Neighbor-joining tree of bHLH TFs in lotus and other species; (B) Neighbor-joining tree of WD40 proteins in lotus and other plants; Numbers close to the nodes indicate bootstrap values, with only values above 50 shown, and scale shows 0.1 amino acid substitutions per site. The deduced amino acid sequences were retrieved from NCBI. Black circles represent lotus bHLH TFs and WD40 proteins that were clustered correspondingly with bHLHs and WD40s of other species known to be involved in the regulation of anthocyanin biosynthesis. All bHLHs and WD40s involved in anthocyanin synthesis were located in the rectangle and labeled as red letters.

Based on the similarity of *bHLHs* among plant species, the full-length cDNA sequences of 10 *bHLHs* were predicted from the genome database of *N. nucifera*. Only two bHLH (NnbHLH1 and NnbHLH2) TFs in *N. nucifera* were clustered together with *Arabidopsis* AtTT8 and petunia PhAN2, which are known to be involved in the regulation of anthocyanins synthesis (Fig. 3A).

From the genome database of *N. nucifera*, the full-length cDNA sequences of four *WD40* genes and partial fragments of thirteen *WD40* genes were identified by local blast. Among the thirteen partial fragments of *WD40* gene, no *WD40* genes were clustered with known WD40s involved in regulating anthocyanins. Thus, only WD40s proteins translated from four full-length cDNA sequences in lotus were aligned with other WD40 proteins from other species to create a phylogenetic tree. The result shows that only NnWD40-1 was clustered with *Arabidopsis* AtTTG1, which is known to be involved in the regulation of anthocyanin synthesis, thereafter NnWD40-1 was further named as NnTTG1 (Fig. 3B).

### Isolation of *NnMYB5, NnbHLH1* and *NnTTG1* genes from red flowers of *N. nucifera*

Specific primers were designed based on the R2R3 domains of nine MYBs and conserved region of two bHLHs, and cDNA of red flowers were used as templates to isolated *MYB* and *bHLH* genes involved in flower coloration. A 289-bp product (GenBank accession: KU198707) was obtained from red flowers. Although many clones were sequenced, all clones only showed the highest identity to *NnMYB5* gene. Only a *bHLH* gene, *NnbHLH1*, was isolated from cDNA of red flowers of *N. nucifera*. Therefore, we inferred that *NnMYB5* and *NnbHLH1* genes may be involved in anthocyanins synthesis in red flowers and further studied.

**Figure 4:**
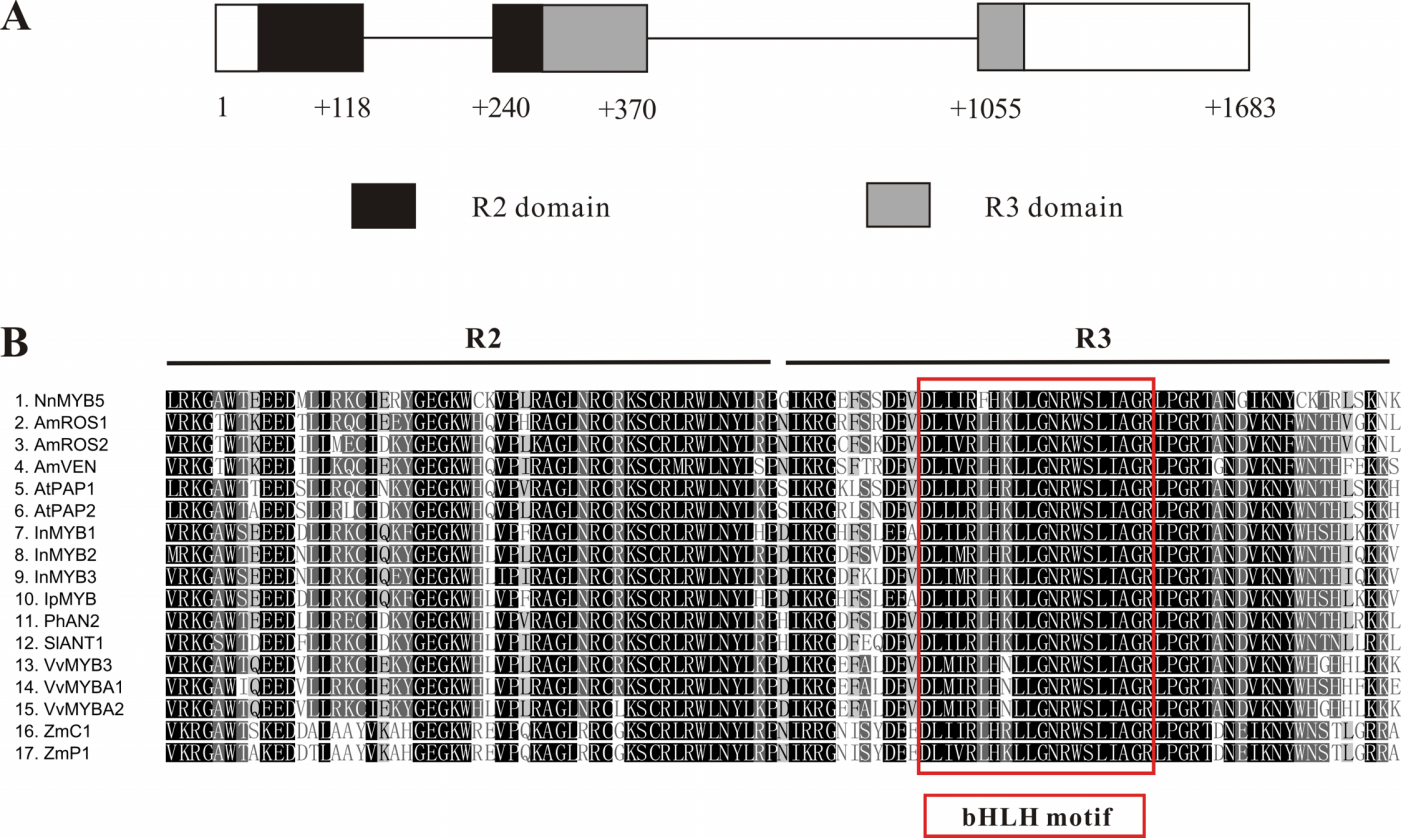
Characterization of *NnMYB5* gene and alignment of NnMYB5 and other anthocyanin-related MYBs from other species. (A) Characterization of *NnMYB5* gene structure. Exons and introns are labeled. Numbers refer to position relative to the first nucleotide of the start codon. Exons are indicated by boxes, and introns are indicated by lines. Location of R2 and R3 domains are highlighted using black and gray backgrounds, respectively. (B) Protein sequence alignment of the R2R3 DNA-binding domains of NnMYB5 and other known anthocyanin MYB regulators in other species. Red rectangle indicates specific residues that interact with bHLH protein. The same residues are evident in NnMYB5, suggesting a protein-protein interaction. Identical residues are shown in black, conserved residues in dark grey and similar residues in light grey.

The 876-bp full-length cDNA sequence of *NnMYB5* (GenBank accession: KU198708) was obtained and its predicted protein comprises 291 amino acids. A comparison between cDNA sequence and the genomic sequence of *NnMYB5* (1,683 bp; GenBank accession: KU198697) revealed that the *NnMYB5* gene comprises three exons and two introns (intron 1 is 122 bp in length, whereas intron 2 is 685 bp long) (Fig. 4A). The R2 domain spans exons 1 and 2, and the R3 domain spans exons 2 and 3. Alignment of the predicted protein sequence of NnMYB5 and the other anthocyanin-related MYB TFs at the R2R3 domain revealed a high degree of homology (Fig. 4B). The NnMYB5 has the amino acid residues ([D/E]Lx_2_[K/R]x_3_ Lx_6_Lx_3_R) that specify interaction with R-like bHLH protein (red box on Fig. 3A). In addition, NnMYB5 also contained three amino acids that are present in the R2 (R instead of G) and R3 domain (V instead of E or D & A instead of E) (Heppel et al., 2013; Kelemen et al., 2015), indicating that NnMYB5 maybe involved in anthocyanin biosynthesis instead of proanthocyanidin. NnMYB5 is closely related to the petunia AN2, with 80.0% amino acid identity over the R2R3 DNA-binding domain and 46.1% identity to the entire protein. For *Arabidopsis* PAP1 and PAP2, these amino acid percentage identities are 78.1, 42.5 and 79, 43.4%, respectively, whilst for other species the overall identities are as follows: grapevine VvMYBA1 43.9%, maize C1 34.5% and maize P1 34.9%. Phylogenetic analysis revealed that NnMYB5 forms a cluster with the other MYB TFs known to be involved in anthocyanin synthesis.

The *NnbHLH1* (GenBank accession: KU198709) comprises a 2,124-bp cDNA region encoding a 707-residue protein. Alignment of the predicted protein sequence of NnbHLH1 and the other anthocyanin-related bHLH TFs at the bHLH domain revealed a high degree of homology (Fig. S2). The phylogenetic tree showed that the NnbHLH1 protein clustered together with petunia AN1 and *Arabidopsis* TT8, with the deduced amino acid sequences exhibiting 56.1 and 42.7% identities, respectively.

The full-length cDNA sequence of *NnTTG1* (GenBank accession: KU198711) comprises a 1,020-bp coding region representing a putative protein of 339 amino acids. *NnTTG1* exhibited high similarity to its homolog from *Arabidopsis* AtTTG1 (77.5% identity at protein-level).

### A comparison of three regulatory genes (*MYB5*, *bHLH1* and *TTG1*) among two species

A comparison of the genomic sequences of *NnMYB5* and *NlMYB5* (GenBank accession: KU198698) revealed forty-three single-nucleotide sequence polymorphisms (SNP), three deletions (3, 2 and 1 bp in size) and three insertions (24, 3 and 46 bp in size) in the coding region, as well as many indels and SNPs in the non-coding region. Among these differences, two nucleotide substitutions (GAA/TAG) in the second exon resulted in a premature stop codon, leading to an incomplete protein in *N. lutea*. By comparison, the promoter of *NlMYB5* (GenBank accession: KU198706) is 107 bp longer than that of *NnMYB5* (GenBank accession: KU198705). In addition, the promoter of *NlMYB5* contained many insertions and SNPs.

Nine amino acids substitutions and a deletion of up to four amino acids were detected in the alignment of the amino acid sequences of NnbHLH1 and NlbHLH1 (GenBank accession: KU198710). Among these differences, one amino acid change occurred in the bHLH domain and four amino acid changes occurred in the N-terminal of bHLH proteins. However, the protein sequence of NlTTG1 (GenBank accession: KU198718) of *N. lutea* is identical to that of NnTTG1 of *N. nucifera*.

### Analysis of *MYB5* gene sequences among populations

**Figure 5:**
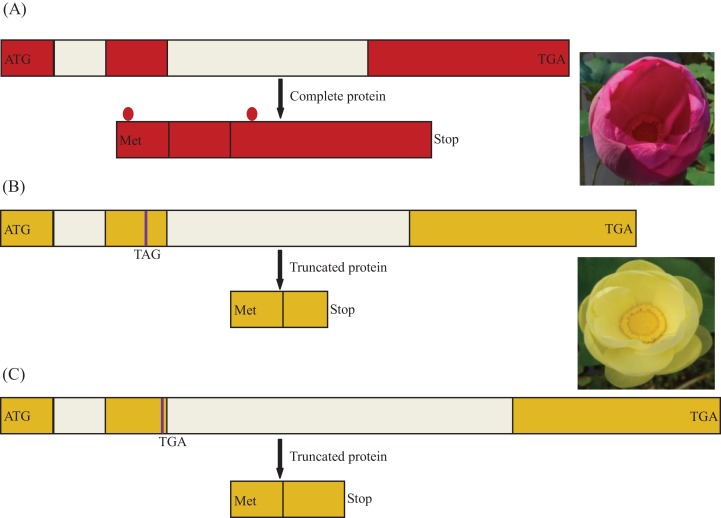
Schema of the *MYB5* allele structures and related encoded proteins in *Nelumbo*. Different colors for the coding regions are reflective of distinct sequence haplotypes. The premature stop codon is indicated in purple line. The region between two red dots is R2R3 motif. (A) A putatively functional form of the *NnMYB5* gene (Genbank accession: KU198697), encoding a complete protein in sacred lotus; (B) Haplotype (Genbank accession: KU198698) with two nucleotide substitutions in second exon of *NlMYB5* gene causing a premature stop codon, resulting in a truncated protein in American lotus; (C) Haplotype (Genbank accession: KU198699) with 1 bp nucleotide deletion in second exon of *NlMYB5* gene causing a frameshift mutation, resulting in a truncated protein in American lotus.

Population analysis allowed the identification of three variants at the *MYB5* locus: (i) the functional allele, encoding a 291 amino acid polypeptide present in the homozygous state in red flowers of *N. nucifera*, (ii) two nucleotide substitutions (GAA/TAG) in the second exon leading to a premature stop codon, (iii) a single-base deletion in the second exon causing a premature stop codon. Remarkably, these two putatively inactive forms of the gene were recognized only in yellow flowers of *N. lutea* (Fig. 5). Nonsense mutations existed in all eight populations, whereas frameshift mutations existed in six populations of eight populations (NLP1 and NLP8). All frameshift mutations coexisted with nonsense mutations as a heterozygous genotype. So far, we did not find homozygous individuals only with framshift mutations.

### Protein–protein interaction of MYB5, bHLH1 and TTG1

**Figure 6:**
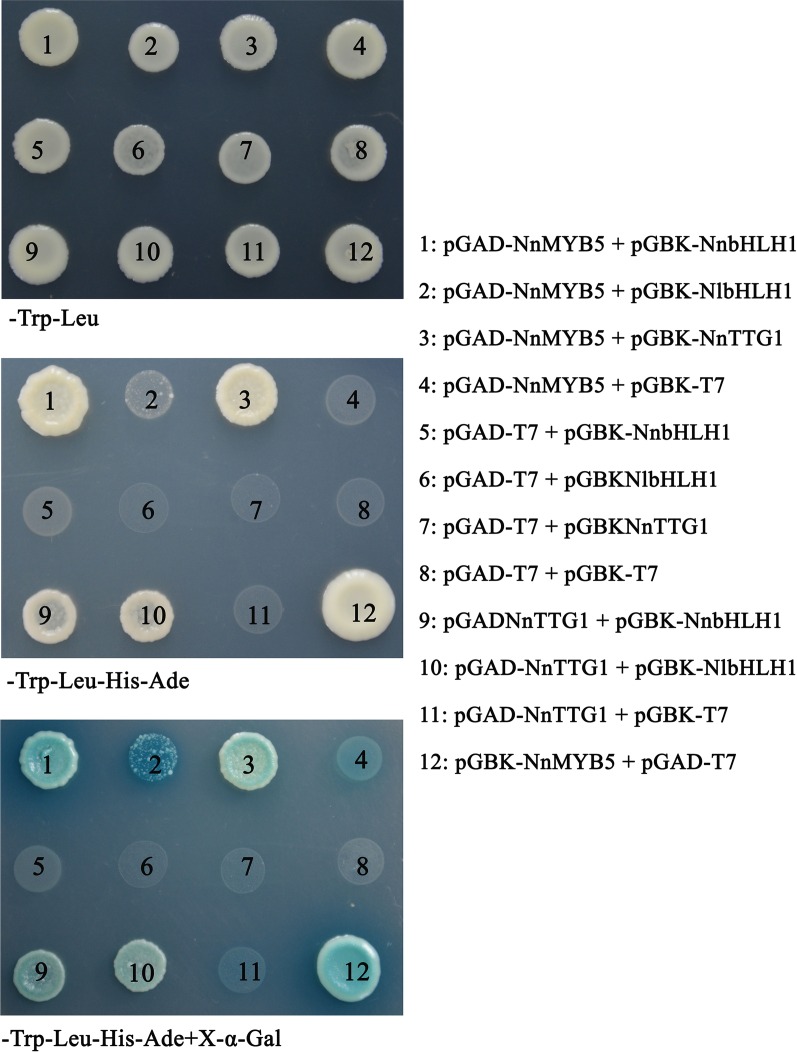
Cooperative interaction of MYB5, NnbHLH1, and NnTTG1 in yeast two-hybrid experiments.

NnMYB5 contains a motif necessary for interactions with R-like bHLH proteins, but no such motif is found in NlMYB5. Thus, whether NnMYB5 interacts with NnbHLH1 or NlbHLH1 was investigated using a GAL4-based yeast two-hybrid system in this study. In addition, the protein-protein interactions between NnTTG1 and other two regulatory factors were also investigated. A construct of the *NnMYB5* gene as a full-length cDNA was tested for transcriptional activation activity in yeast in this study and the result showed that the NnMYB5 has transcriptional activity in yeast (Fig. 6). Thus, the interaction study was carried out with DNA-binding domain-fused NnbHLH1 and NnTTG1, and activation domain-fused NnMYB5 and NnTTG1. Only the yeast harboring the combination of NnMYB5-NnbHLH1, NnMYB5-NnTTG1, NnTTG1-NnbHLH1, NnMYB5-NlbHLH1 and NnTTG1-NlbHLH1 survived on quadruple dropout medium, and their color turned to blue when grown on SD–Trp–Leu–His–Ade + X–α-Gal media (Fig. 6). This suggests that NnMYB5 interacts with NnbHLH1, NlbHLH1 and NnTTG1, as well as the interaction between NnTTG1 and NnbHLH1 and NlbHLH1.

### Functional test of *NnMYB5* in transgenic plants

**Figure 7:**
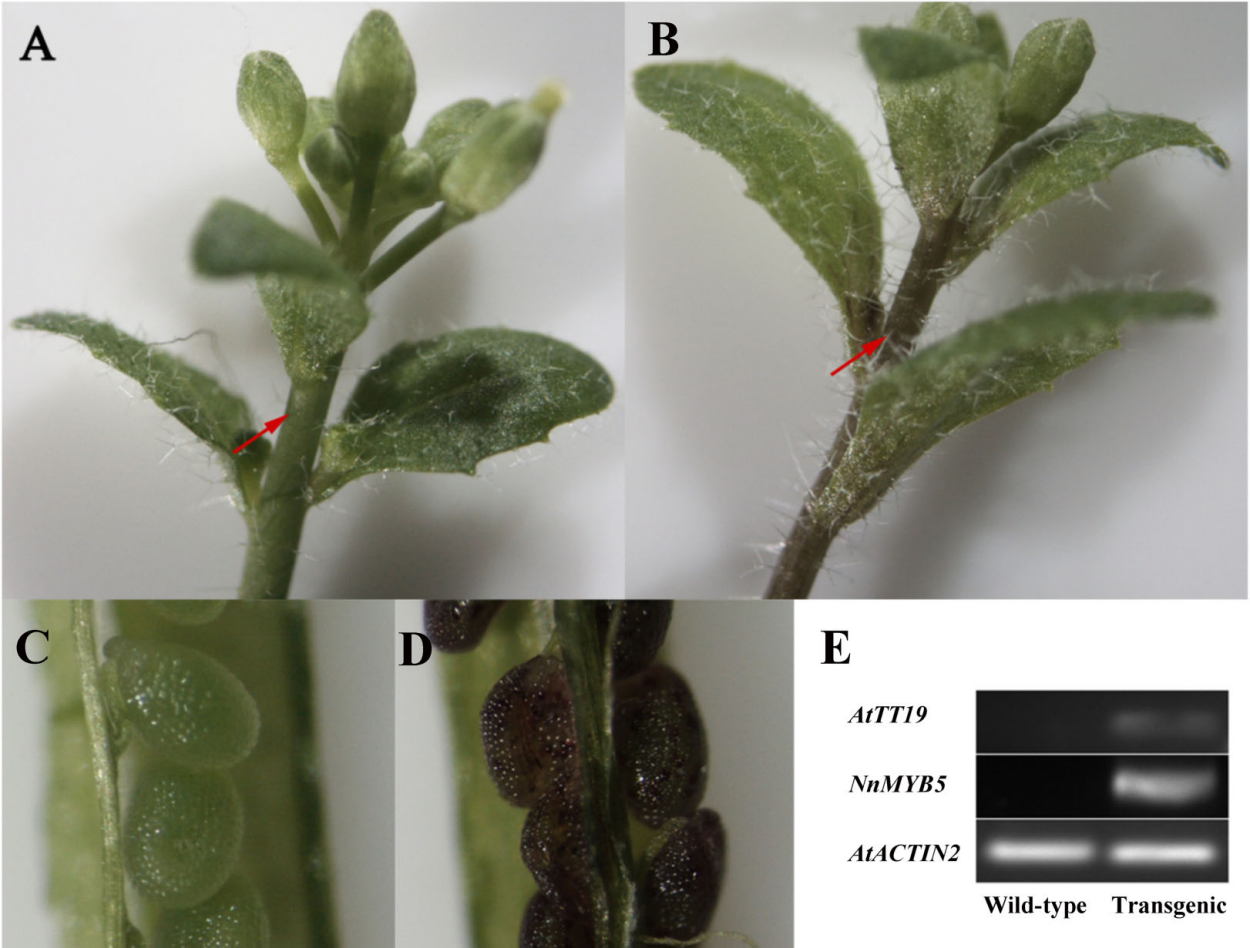
Functional analysis of *NnMYB5* in transgenic plants. Photographs of flower stalks from *Arabidopsis* ecotype Columbia: (A) untransformed plants and (B) transformed with *NnMYB5* DNA. Arrow indicates accumulated anthocyanins in flower stalks. Photographs of immature seeds (less than 10 d after anthesis) from *Arabidopsis* ecotype Columbia: (C) untransformed plants and (D) transformed with *NnMYB5* DNA. (E) The expression levels of *NnMYB5* and *AtTT19* in wild-type and transgenic plants as determined by semi-quantitative RT-PCR. *AtACTIN2* gene was used as the internal control.

To test the function of *NnMYB5*, we constructed the expression vector of *NnMYB5* with CaMV 35S promoter and transformed it into *Arabidopsis* plants. Some seeds collected from Hygromycin-resistant T1 plants were brown, which were similar to the seeds of wild-type plants. However, other seeds of T1 plants were purple in color. Although the brown seeds of T1 plants had a higher seed germination rate in soil than purple seeds, almost all the seedlings that germinated from brown seeds were not transgenic plants. Most of transgenic T2 seedlings showed no visible pigmentation anywhere in the entire plant. Only a few transgenic T2 seedlings showed pale purple in flower stalks. Under a stereomicroscope, there was clear ectopic pigmentation in immature seeds (< 10 days after anthesis) and flower stalks of transgenic plants, but not in immature seeds and flower stalks in wild-type control plants (ecotype Columbia) (Figs. 7A and 7B). Our sqRT–PCR results suggested that *NnMYB5* was only expressed in the transgenic plants (Fig. 7C). Additionally, sqRT-PCR results also showed that the expression level of *TT19* gene was up-regulated in *NnMYB5* over-expression plants.

## Discussion

The variation of regulatory genes in the anthocyanin biosynthetic pathway could contribute to the diversity of plant flower color (Nakatsuka et al., 2005). In this study, three transcriptional genes were isolated from cDNAs of red flowers, named *NnMYB5*, *NnbHLH1* and *NnTTG1*, respectively. Our results demonstrated that the above three TF can mutually interact, and MYB5 is a transcriptional regulator of anthocyanin synthesis. Finally, we found a relationship between *MYB5* gene and flower color difference of two species in *Nelumbo*.

### MYB5, bHLH1, and TTG1 can interact simultaneously

The complex composed of three types of regulatory genes (*R2R3-MYB*, *bHLH* and *WD40*) activates transcription of flavonoid biosynthetic genes and thus regulates anthocyanin synthesis (Koes, Verweij & Quattrocchio, 2005; Ramsay & Glover, 2005). In this study, three regulatory genes (*NnMYB5*, *NnbHLH1* and *NnTTG1*) involved in anthocyanin synthesis were isolated from red flowers. It is well known that R2R3-MYBs interact with bHLHs, such as the interaction between MrbHLH1 and MrMYB1 in bayberry and GtbHLH1 interacting with GtMYB3 in gentian (Nakatsuka et al., 2008; Liu et al., 2013). In this study, NnMYB5 interacts with NnbHLH1 or NlbHLH1. It is uncertain whether WD40 can interact with MYB. For example, in *Arabidopsis*, TTG1 (WD40) can interact with TT2; in apple, MdTTG1 interacts with bHLH but not MYB proteins (An et al., 2012). In *Nelumbo*, NnTTG1 not only interacts with two bHLHs (NnbHLH1 and NlbHLH1) but also interacts with NnMYB5. The interaction of NlbHLH1-NnMYB5 was weaker than the interaction of NnbHLH1-NnMYB5, which indicated that amino acid differences between NlbHLH1 and NnbHLH1 affected the interaction with NnMYB5.

### NnMYB5 is a transcriptional activator in anthocyanin biosynthesis

Amino acid sequence similarity, protein structures, and phylogenetic analysis suggested that NnMYB5 may exhibit a similar function to other MYB homologues regulating anthocyanin synthesis. However, methods for genetic transformation of lotus have not been established. Therefore, we introduced the full-length gene of *NnMYB5* into *Arabidopsis* plants in order to test the function of *NnMYB5*. The transgenic plants developed partially red seeds and purple pedicels, indicating that NnMYB5 can function as a transcriptional activator in anthocyanin biosynthesis. This result is consistent with the regulatory gene *MdMYB1* in red apple and *RsMYB1* in radish (Takos et al., 2006; Lim et al., 2016).

MYB TFs lead to anthocyanin accumulation by up-regulating the transcripts of some structural genes (Tohge et al., 2005). For example, in tomato, over-expression of *AN1*(*MYB*) led to up-regulation of *GST* (Mathews et al., 2003); in *Arabidopsis*, the expression of *TT19* (*GST*) is up-regulated in the *PAP1* over-expressing plants (Tohge et al., 2005); in apple, *MdMYB10* activates the transcription of *DFR* (Espley et al., 2007); in grape, the ectopic expression of *VvMYB1* activates the expression of *UFGT* and *GST* (Cutanda-Perez et al., 2009); in nectarine, MYB10 positively regulates the promoters of *UFGT* and *DFR* (Ravaglia et al., 2013); in ever-red leaf crabapple, *McMYB* activates *McF3′H* and later structural genes (Tian et al., 2015). Due to the lack of *GST* transcripts and inactivation of *MYB5* simultaneously occurred in yellow flowers of *N. lutea*, so we investigated whether NnMYB5 can regulate the expression of *GST* genes in transgenic *Arabidopsis*. The results showed that the expression of *TT19* (*TT19*) was up-regulated in *NnMYB5* over-expressing plants. Therefore, we inferred that MYB5 transcriptional factor may regulates the expression level of *GST* in *Nelumbo*. The direct activation of MYB5 on *NnGST* needs to be investigated. In addition, the expression of *DFR* and *ANS* in yellow flower is higher than that of red flowers, indicating that they maybe controlled by other *MYB* instead of MYB5.

### A relationship between flower color difference of two species in *Nelumbo* and *MYB5* gene

To date, most of previous studies on the genetic basis of flower coloration suggest that variation in regulatory genes is central to variation in pattern and intensity of pigmentation (Schwinn et al., 2006; Yamagishi et al., 2010). Due to the high specificity of *MYB* in regulating anthocyanin biosynthetic pathway, mutations in *MYB* genes are expected to incur the least pleiotropic effects and are most likely to be involved in the evolution of flower color (Schwinn et al., 2006; Streisfeld & Rausher, 2011). For example, in *Petunia axillaris*, the absence of anthocyanins in white flowers is closely related to loss of *AN2* function (Quattrocchio et al., 1999; Hoballah et al., 2007); in *Mimulus*, gains of pigmentation in petal lobes are attributable to the evolution of R2R3-MYB TF (Cooley et al., 2011); in *Antirrhinum*, variation in three *R2R3-MYB* regulatory genes might account for the flower color variation among species (Schwinn et al., 2006); in *Oncidium*, the red/yellow color of floral lip depends on whether *OgMYB1* is expressed or not (Chiou & Yeh, 2008); in apple, differential expression of *MYB10* led to differences in anthocyanin levels between red and green strips (Telias et al., 2011); in gentian, the loss of functional *GtMYB3* expression leads to the absence of anthocyanin in white flower (Nakatsuka et al., 2008). In this study, a striking difference in *MYB5* gene was detected in two lotus species. In all populations of *N. lutea* with yellow flowers, *MYB5* is a nonfunctional gene; however, in all populations of *N. nucifera* with red-flowers, *MYB5* is a functional gene. It indicated a relationship between flower color difference and loss of functional *MYB5* gene.

## Conclusions

In this study, three regulatory genes (*NnMYB5*, *NnbHLH1* and *NnTTG1*) involved in anthocyanin synthesis were identified from red flower of *N. nucifera*. Our investigations revealed that MYB5 is a functional transcription activator of anthocyanin synthesis. The flower color difference between red flowers and yellow flowers may be related to the variation of *MYB5* gene. The exact of flower color difference needs further investigation.

## Supplemental Information

10.7717/peerj.2369/supp-1 Supplemental Information 1 Phylogenetic tree showing relationships between the 92 lotus MYB TFs with MYBs of other species. Numbers close to the nodes indicate bootstrap values, with only values above 50 shown, and scale shows 0.1 amino acid substitutions per site. Black circles represent lotus MYB TFs that were clustered MYBs with other species known to be involved in the regulation of anthocyanin biosynthesis.

10.7717/peerj.2369/supp-2 Supplemental Information 2 Alignment of bHLH domain of NnbHLH1 and other anthocyanin-related bHLHs from other species. Identical residues are shown in black, conserved residues in dark grey and similar residues in light grey.

10.7717/peerj.2369/supp-3 Supplemental Information 3 Details of sample locations, sample size (n) and haplotypes of 16 populations of *N. lutea* (NlP1-8) and *N. nucifera* (NnP1-8) surveyed for DNA sequence variation in the second exon of *MYB5* gene. tru.

10.7717/peerj.2369/supp-4 Supplemental Information 4 Gene-specific primers used in this study.

10.7717/peerj.2369/supp-5 Supplemental Information 5 GenBank accessions of all contigs used for the identification of 95 MYB, 10 bHLH and 17 WD40 transcription factors in lotus genome.

## List of Abbreviations

ANS: anthocyanidin synthase
bHLH: basic helix-loop-helix
DFR: dihydroflavonol-4-reductase
GST: glutathione S-transferase
ORF: open reading frame
qRT-PCR: quantitative real-time polymerase chain reaction
sqRT-PCR: semi-quantitative real-time polymerase chain reaction
TF: transcription factor
UFGT: UDP-glucose: flavonoid-3-O-glucosyltransferase.

## Additional Information and Declarations

### DNA Deposition

The following information was supplied regarding the deposition of DNA sequences: GenBank accession no. KU198705 to KU198711; GenBank accession no. KU198718; GenBank accession no. KU198697 to KU198699; GenBank accession no. KU198671 to KU198695.

### Data Deposition

The following information was supplied regarding data availability: Sun, Shan-Shan (2016): Data S1. Figshare: https://dx.doi.org/10.6084/m9.figshare.3370837.v1.
