# Supplementary figures and images for: Identification of a R2R3-MYB gene regulating anthocyanin biosynthesis and relationships between its variation and flower color difference in lotus (Nelumbo Adans.)
Source: PeerJ. 2016 Sep 1;4:e2369. doi: 10.7717/peerj.2369 (PMC5012265; doi:10.7717/peerj.2369)

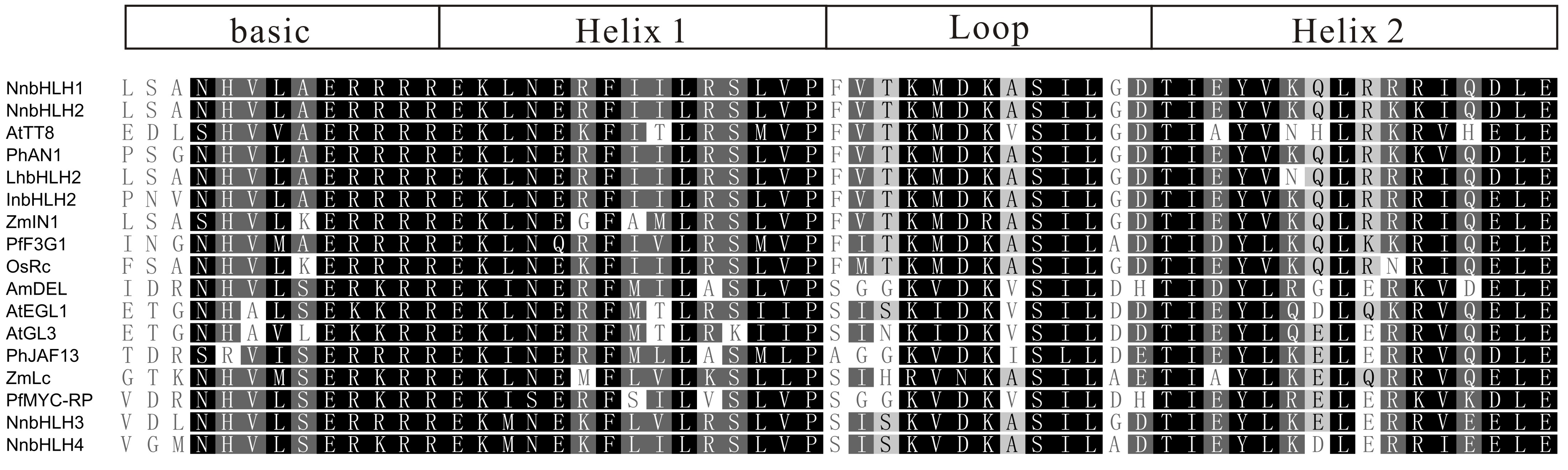

Supplement: Supplemental Information 2 — Identical residues are shown in black, conserved residues in dark grey and similar residues in light grey. [file peerj-04-2369-s002.png]
