# Supplementary material for: Identification of a R2R3-MYB gene regulating anthocyanin biosynthesis and relationships between its variation and flower color difference in lotus (Nelumbo Adans.)
Source: PeerJ. 2016 Sep 1;4:e2369. doi: 10.7717/peerj.2369 (PMC5012265; doi:10.7717/peerj.2369)
Supplement: Supplemental Information 3 [file peerj-04-2369-s003.docx]

**Table S1.**

| Population | Location | Coordinates (E/N) | n | Haplotypes |
| --- | --- | --- | --- | --- |
| NlP1 | Lake Waccamaw NC, USA | -78°30’/34°18’ | 6 | H1 |
| NlP2 | Small Wood State Park. MD, USA | -77°12’/38°30’ | 6 | H2,H3,H13,H14 |
| NlP3 | Lotus Covo, MS, USA | -87°48’/34°15’ | 7 | H2,H4,H5,H15 |
| NlP4 | Gantt, W. HWY, AL, USA | -86°30’/31°24’ | 6 | H2,H6,H7,H16 |
| NlP5 | Lake Murray State Park, OK, USA | -97°06’/34°04’ | 6 | H1,H8,H9,H17 |
| NlP6 | Stephen A. Forbes State Park, IL, USA | -88°42’/38°42’ | 6 | H2,H4,H10,H11,H18 |
| NlP7 | Pymatuning Lake, PA, USA | -80°24’/41°18’ | 7 | H1,H8 |
| NlP8 | Jackson Lake, FL, USA | -85°12’/30°42’ | 4 | H2,H12,H13,H14 |
| NnP1 | Mudanjiang, Heilongjiang | 129°39’/44°03’ | 6 | H19 |
| NnP2 | Yilan, Heilongjiang | 129°35’/46°04’ | 6 | H19 |
| NnP3 | Wuchang, Heilongjiang | 127°10’/46°05’ | 6 | H19 |
| NnP4 | Jixi, Heilongjiang | 130°49’/45°01’ | 6 | H19 |
| NnP5 | Thailand | 99°7’/9°33’ | 6 | H19, H20,H21 |
| NnP6 | Thailand | 103°7’/14°59’ | 6 | H19,H22 |
| NnP7 | Thailand | 102°43’/14°59’ | 6 | H19,H23 |
| NnP8 | Thailand | 100°36’/14°11’ | 7 | H19,H24,H25 |

**Note:** twenty-five haplotypes (H1- H25) were deposited in GenBank under accession nos. KU198671 to KU198695.
