# Supplementary material for: Identification of a R2R3-MYB gene regulating anthocyanin biosynthesis and relationships between its variation and flower color difference in lotus (Nelumbo Adans.)
Source: PeerJ. 2016 Sep 1;4:e2369. doi: 10.7717/peerj.2369 (PMC5012265; doi:10.7717/peerj.2369)
Supplement: Supplemental Information 4 [file peerj-04-2369-s004.docx]

**Table S2**.

| Primer name | Sequence(5′-3′) |  | |
| --- | --- | --- | --- |
| MYBU1F  MYBU1R  MYBU2F1  MYBU2R1  MYBU2F2  MYBU2R2  MYBU3F  MYBU3R  bHLH205F  bHLH1420R  MYB5gR  bHLHgF  bHLHgR  TTG1gF  TTG1gR  yeastMYB5F-AD  yeastMYB5F-BK  yeastMYB5R  yeastbHLH1F  yeastbHLH1R  yeastTTG1F  yeastTTG1R  MYB1pr-F  MYB1pr-R  RTMYB-F  RTMYB-R  RTAtGST12-F  RTAtGST12-R  RTAtactin-F  RTAtactin-R  RTGST-F  RTGST-R  RTactin-F  RTactin-R  RTDFR-F  RTDFR-R  RTANS-F  RTANS-R  RTUGT1-F  RTUGT1-R  RTUGT2-F  RTUGT2-R  RTUGT3-F  RTUGT3-R | ATGGATGGTGGTTTGGGTTTGAGAA  GCTGTTCTTCCGGGAAGTCT  ATGGAGGGTCGTTCTTTGGGTTT  CTACAGCTCTTTCGACATCTCCG  ACGGAGATGTCGAAAGAGCTGTA  TGTGGAGCCTAAATATGAGATCAG  GGTTGCTGGACTGAAGAAGAAGAT  SYGCGWATTSCAGTAGTTCTTRA  AGTCAACAGCTTAGGGAACTCTACG  CCGCCGTTCTGCTAAGACATG  CACCTGACGGATTTTATTGGGCTTC  CTTGAAGCACTCGCAGAGACGAAT  AAGCCACGGTTCATTCTCTTATCT  TCAGCAGAGCCGTCACGATATTAGC  AACTGCCAACACTCAAGACATATCG  CCGGAATTC**ATG**GATGGTGGTTTGGGTTT  CATGCCATGG**ATG**GTGGTTTGGGTTT  CGCGGATCC**TCA**ATAACTCCACCACCTATG  CATGCC**ATG**GCTACCCCAGGGAGTAA  CGCGGATCC**TCA**GTATTGATTTATGATTTGGTGTTA  CCGGAATTC**ATG**GATAATTCCACCCAAG  CGCGGATCC**TCA**AACCTTCAGAAGCTGC  CTTTACATTCCGTTATTGTTTCCCTC  TTGCCTTCTCCATACCTCTCAATGC  GACAACTCCTGATTTTCCACCGCCTA  TCATTTCTGACGCTAATAAGTCCCAA  GAACCTTTTGGGCAAGTCTCTAGAGC  TGTGCGTCAAATCAGCCATAGTGAAT  GGTAACATTGTGCTCAGTGGTGG  AACGACCTTAATCTTCATGCTGC  GTGGAGTTCGAAGTAATACATG CATTCGTGGGAGGATGAGTAGTTC  AUGCCCUGAUGAAGAUCCUUA  TCAAGGATGGCTGGAATAGAACCTCA  GTGATAAAGCCATGTATCGAGGGTGT  ACATGATAAAAGGCCCAACTACAAGC  AAGCGTGATTTGTCTATGTGGCCCAA  GCTTCAACGCCAAGTGCTAGATCCG  AACCTCACACGTAGACTAGCAAC  CCTCCGTCATCTCTGCCGCGAAGTAT  ACTACACTGATTTCATCCGCCACA  TCCAGCTCCCGAAAGGAATTAACGA  AAGCCTATGATATCTCAGACGGAATC  GCAGGTGATATTCTTCCCCGTCT | | Used for the identification of regulatory genes from red flowers  Used for the full-length sequence of corresponding genes  Used for Y2H system  Used for obtaining promoter region  Used for sqRT-PCR  Used for qRT-PCR |

**Notes:** The restriction sites are underlined. The full-length of *MYB5* was ligated with pGADT7 and pGBKT7 vector, so the forward primers were named as yeastMYB5F-AD and yeastMYB5F-BK, respectively. Bold letters respresent start and stop codons.
