# Supplementary material for: Identification of a R2R3-MYB gene regulating anthocyanin biosynthesis and relationships between its variation and flower color difference in lotus (Nelumbo Adans.)
Source: PeerJ. 2016 Sep 1;4:e2369. doi: 10.7717/peerj.2369 (PMC5012265; doi:10.7717/peerj.2369)
Supplement: Supplemental Information 5 [file peerj-04-2369-s005.docx]

**Table S3.**

| Gene | Genbank Accession No. |
| --- | --- |
| NnMYB1 | AQOG01018314.1 |
| NnMYB2 | AQOG01033025.1 |
| NnMYB3 | AQOG01018863.1 |
| NnMYB4 | AQOG01000706.1 |
| NnMYB5 | AQOG01026491.1 |
| NnMYB6 | AQOG01033433.1 |
| NnMYB7 | AQOG01017596.1 |
| NnMYB8 | AQOG01000161.1 |
| NnMYB9 | AQOG01020708.1 |
| NnMYB10-1 | AQOG01001540.1 |
| NnMYB10-2 | AQOG01001540.1 |
| NnMYB10-3 | AQOG01001540.1 |
| NnMYB11 | AQOG01026505.1 |
| NnMYB12 | AQOG01026490.1 |
| NnMYB13 | AQOG01028619.1 |
| NnMYB14 | AQOG01003228.1 |
| NnMYB15 | AQOG01018106.1 |
| NnMYB16-1 | AQOG01026488.1 |
| NnMYB16-2 | AQOG01026488.1 |
| NnMYB17 | AQOG01013411.1 |
| NnMYB18 | AQOG01013412.1 |
| NnMYB19-1 | AQOG01025278.1 |
| NnMYB19-2 | AQOG01025278.1 |
| NnMYB20 | AQOG01012433.1 |
| NnMYB21 | AQOG01025279.1 |
| NnMYB22 | AQOG01025826.1 |
| NnMYB23 | AQOG01020038.1 |
| NnMYB24 | AQOG01004186.1 |
| NnMYB25 | AQOG01016608.1 |
| NnMYB26 | AQOG01001607.1 |
| NnMYB27 | AQOG01009837.1 |
| NnMYB28 | AQOG01033024.1 |
| NnMYB29 | AQOG01028325.1 |
| NnMYB30 | AQOG01033017.1 |
| NnMYB31 | AQOG01033802.1 |
| NnMYB32 | AQOG01038157.1 |
| NnMYB33 | AQOG01005290.1 |
| NnMYB34 | AQOG01001779.1 |
| NnMYB35 | AQOG01018258.1 |
| NnMYB36 | AQOG01012503.1 |
| NnMYB37 | AQOG01009412.1 |
| NnMYB38 | AQOG01001273.1 |
| NnMYB39 | AQOG01031421.1 |
| NnMYB40 | AQOG01015939.1 |
| NnMYB41 | AQOG01030630.1 |
| NnMYB42-1 | AQOG01013413.1 |
| NnMYB42-2 | AQOG01013413.1 |
| NnMYB43 | AQOG01018530.1 |
| NnMYB44 | AQOG01050923.1 |
| NnMYB45 | AQOG01009647.1 |
| NnMYB46 | AQOG01013507.1 |
| NnMYB47 | AQOG01015676.1 |
| NnMYB48 | AQOG01035142.1 |
| NnMYB49 | AQOG01026478.1 |
| NnMYB50 | AQOG01031793.1 |
| NnMYB51 | AQOG01006984.1 |
| NnMYB52 | AQOG01002201.1 |
| NnMYB53 | AQOG01044897.1 |
| NnMYB54 | AQOG01010841.1 |
| NnMYB55 | AQOG01029299.1 |
| NnMYB56 | AQOG01029111.1 |
| NnMYB57**^*^** | AQOG01013632.1 |
| NnMYB58-1 | AQOG01020227.1 |
| NnMYB58-2 | AQOG01020227.1 |
| NnMYB59 | AQOG01015873.1 |
| NnMYB60 | AQOG01000971.1 |
| NnMYB61 | AQOG01004704.1 |
| NnMYB62 | AQOG01032840.1 |
| NnMYB63 | AQOG01016223.1 |
| NnMYB64 | AQOG01013851.1 |
| NnMYB65 | AQOG01024540.1 |
| NnMYB66 | AQOG01010265.1 |
| NnMYB67 | AQOG01024690.1 |
| NnMYB68^*^ | AQOG01000955.1 |
| NnMYB69 | AQOG01010291.1 |
| NnMYB70 | AQOG01029112.1 |
| NnMYB71-1 | AQOG01028519.1 |
| NnMYB71-2 | AQOG01028519.1 |
| NnMYB72 | AQOG01034196.1 |
| NnMYB73 | AQOG01041111.1\| |
| NnMYB74 | AQOG01039353.1 |
| NnMYB75^*^ | AQOG01042094.1 |
| NnMYB76 | AQOG01026502.1 |
| NnMYB77 | AQOG01018588.1 |
| NnMYB78 | AQOG01012120.1 |
| NnMYB79 | AQOG01028758.1 |
| NnMYB80 | AQOG01006636.1 |
| NnMYB81 | AQOG01004747.1 |
| NnMYB82 | AQOG01021893.1 |
| NnMYB83 | AQOG01022336.1 |
| NnMYB84 | AQOG01012944.1 |
| NnMYB85 | AQOG01042099.1 |
| NnMYB86 | AQOG01030670.1 |
| NnMYB87 | AQOG01042096.1 |
| NnMYB88  NnbHLH1  *NnbHLH2*  NnbHLH3  NnbHLH4  NnbHLH5  NnbHLH6  NnbHLH7  NnbHLH8  NnbHLH9  NnbHLH10  NnWD40-1 (NnTTG1)  NnWD40-2  NnWD40-3  NnWD40-4  NnWD40-5  NnWD40-6  NnWD40-7  NnWD40-8  NnWD40-9  NnWD40-10  NnWD40-11  NnWD40-12  NnWD40-13  NnWD40-14  NnWD40-15  NnWD40-16  NnWD40-17 | AQOG01034559.1  AQOG01008515.1  AQOG01002332.1  AQOG01007910.1  AQOG01034656.1  AQOG01024603.1  AQOG01027389.1  AQOG01035295.1  AQOG01000883.1  AQOG01018784.1  AQOG01005821.1  AQOG01000638.1  AQOG01004307.1  AQOG01022925.1  AQOG01010670.1  AQOG01009782.1  AQOG01019684.1  AQOG01026554.1  AQOG01024604.1  AQOG01012020.1  AQOG01025845.1  AQOG01046228.1  AQOG01003717.1  AQOG01030211.1  AQOG01001212.1  AQOG01035001.1  AQOG01032126.1  AQOG01002889.1 |

**Note:** * represent that the R2R3 motifs were not discovered from corresponding contigs, so these three genes (*NnMYB57*, *NnMYB*68 and *NnMYB75*) are not involved in phylogenetic analysis.
